# First record of *Neolindus* Scheerpeltz from French Guiana (Coleoptera, Staphylinidae, Paederinae), with a key to males

**DOI:** 10.3897/zookeys.135.1740

**Published:** 2011-10-07

**Authors:** Angélico Asenjo

**Affiliations:** 1 Laboratório de Sistemática e Bioecologia de Coleoptera (Insecta), Departamento de Zoologia, Universidade Federal do Paraná, Caixa Postal 19020, 81531–980, Curitiba, Paraná, Brazil

**Keywords:** Coleoptera, Staphylinidae, Paederinae, *Neolindus*, key, French Guiana, new species

## Abstract

The genus *Neolindus* Scheerpeltz, 1933, of the tribe Cylindroxystina Bierig, 1943, is recorded from French Guiana for the first time. Two new species, *Neolindus irmleri* **sp. n.** and *Neolindus hermani* **sp. n.**, are described and illustrated. A key to males of *Neolindus* is provided.

## Introduction

The genus *Neolindus*Scheerpeltz, 1933 was revised by Herman (1991) in his extensive phylogenetic work and revision of the tribe Cylindroxystina. In that publication he described three characters that support the monophyletic status of the genus and described 27 species, principally from South America.

More recently, Irmler (2011) described two new species from the Peruvian Amazon. *Neolindus*is currently the larger of the two Neotropical genera (*Neolindus* and *Cylindrxystus*) of Cylindroxystina, represented by 35 species distributed from northern Costa Rica to southern Brazil (Herman 2001, Irmler 2011), 29 of which are known only from South America. *Neolindus* species are typically found in forest leaf litter at altitudes ranging from 50 to 2500 m.

In this paper, two new species of *Neolindus* are described and illustrated and the genus is recorded for the first time from French Guiana, increasing the number of known species to 37. A key to males of the genus is presented based mainly on characters of the genitalia. *Neolindus amazonicus* Irmler 1981, *Neolindus peruvianus* Irmler 1981 and *Neolindus hanagarthi* Irmler 1981 are excluded from the key because they are known only from females.

## Methods

**Specimens.** Specimens were collected via flight interception traps (FIT or window traps), an especially useful capture method which has resulted in the discovery of many new species of multiple taxa. The specimens studied here were collected by SEAG (Société Entomologique Antilles-Guyane) with FITs hung approximately 1.50 m above ground level (Fig. 1 in Degallier et al. 2011). Traps consisted of an acrylic panel hung vertically in the forest and a gutter at the base of the panel containing a preservative fluid (water, salt and detergent, or propylene glycol).

To study morphological characters, dried specimens were macerated in boiling water for five minutes and then cleared in 10% KOH overnight. Dissections were carried out under a Carl Zeiss Stemi SV6 stereoscopic microscope and drawings made with the same equipment. Photographic illustrations were done using IM 50 (Image Manager) software and combined using Auto-Montage Pro (Syncroscopy) software. Measurements were made with an ocular micrometer in the SV6 microscope.

For the type label data, quotation marks “ ” separate different labels and a slash / separates different lines. Text within square brackets [ ] is explanatory and was not included in the original labels.

The following abbreviations are used:

BL	body length (from anterior margin of clypeus to posterior margin of tergite IX)

BW	body width (maximum width of elytra)

EL	elytral length (maximum)

EW	elytral width (maximum)

HL	head length (from anterior margin of clypeus to posterior margin of head disc)

HW	head width (maximum)

PL	pronotum length (maximum)

PW	pronotum width (maximum)

All measurements are in millimeters and are based on the holotypes. The terminology adopted for the descriptions follows (Irmler (1981, 2011) and Herman (1991).

## Depositories

All specimens are deposited in the following collections:

**DZUP	**Coleção de Entomologia Pe. J. S. Moure, Departamento de Zoologia, Universidade Federal do Paraná, Curitiba, Brazil (Lucia M. de Almeida).

**MNHN	**Muséum National d’Histoire Naturelle, Paris, France (Thierry Deuve).

**MUSM**	Colección Entomológica del Museo de Historia Natural de la Universidad Nacional Mayor de San Marcos, Lima, Peru (Gerardo Lamas).

## Results

### Neolindus irmleri

Asenjo sp. n.

urn:lsid:zoobank.org:act:DF665432-A4A7-4173-B61B-166F17AADBFD

http://species-id.net/wiki/Neolindus_irmleri

Figs 1–7 

#### Type material.

FRENCH GUIANA: Holotype male, with labels: “GUYANA FRANCESA: / Montagne des chevaux, / 04°43'N, 52°25'W, 90m, / flight intercept trap(glass), / 9.v.2009, S. Brûlé, / P.H. Dalens, E. Poirier” “Holotype / *Neolindus* / *irmleri* Asenjo / Desig. Asenjo, 2011” (MNHN).

#### Paratype.

1 malewith labels: “GUYANA FRANCESA: / Montagne des chevaux, / 04°43'N, 52°25'W, 90m, / flight intercept trap(glass), / 13.vi.2009, S. Brûlé, / P.H. Dalens, E. Poirier” “Paratype / *Neolindus* / *irmleri* Asenjo / Desig. Asenjo, 2011” (DZUP).

#### Diagnosis.

 *Neolindus irmleri* sp. n. can be distinguished from other *Neolindus* species by the sternum VIII divided into one central and two lateral plates (Fig. 7).

#### Description.

Holotype male, BL: 12.36.

**Figures 1–7.**
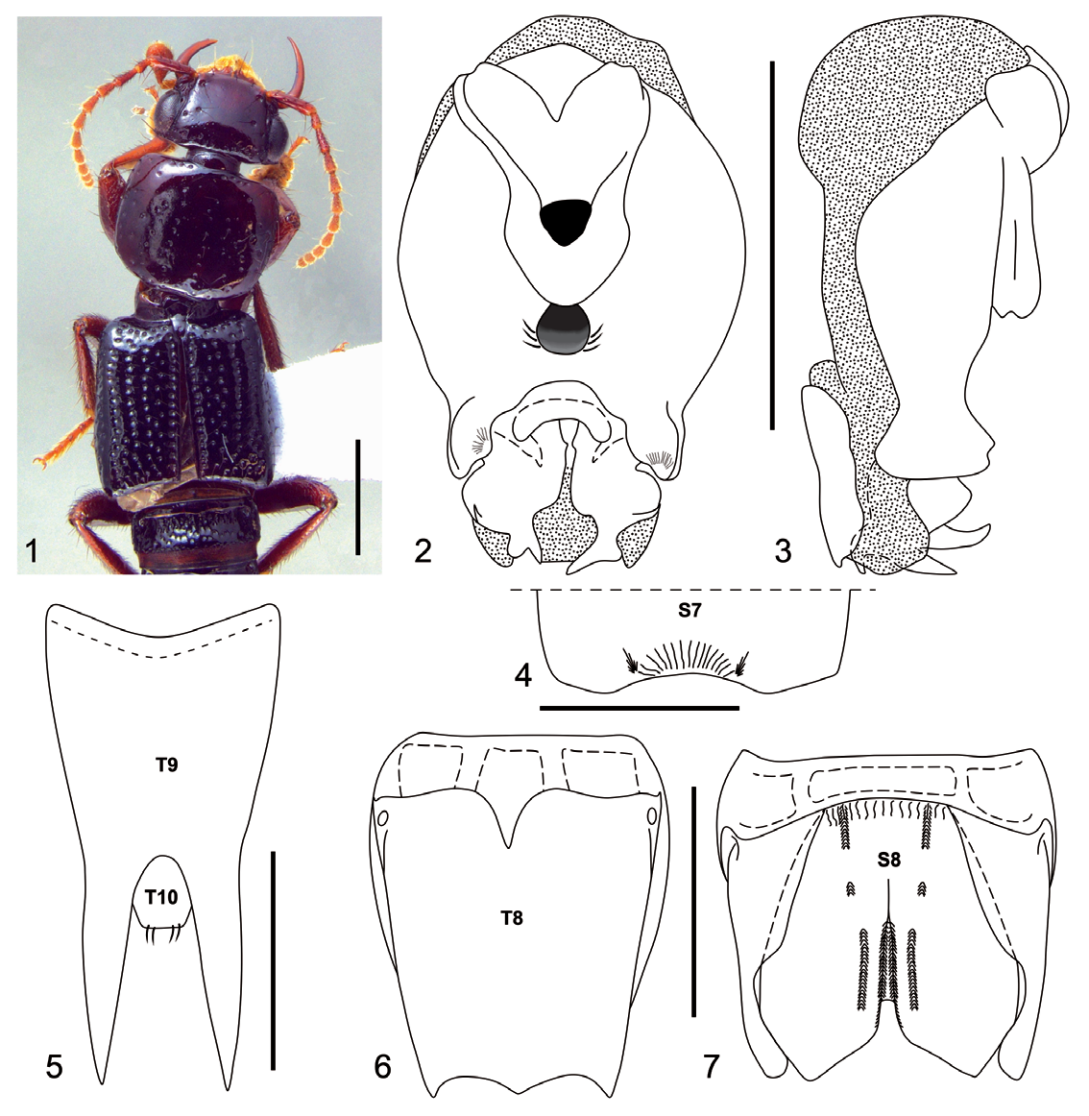
*Neolindus irmleri* Asenjo sp. n. holotype male. **1** Adult habitus **2** aedeagus, ventral view **3 **aedeagus, lateral view **4** apex of sternum VII (S7), setae omitted **5** tergum IX (T9) and tergum X (T10), setae omitted **6** tergum VIII (T8), setae omitted **7** sternum VIII (S8), setae omitted. Scale bars= 1mm.

Body dark brown (Fig. 1). Mandibles, femora and tibiae dark reddish brown; antennal segments 1–3 dark reddish brown, segments 4–11 and all tarsi paler.

Head and pronotum moderately flattened dorsoventrally. Head (Fig. 1) wider (HW: 1.61) than long (HL: 1.02), with acute hind angles. Head disk with umbilicate punctures each carrying a black macroseta and one trichobothrium on lateral side of vertex near anterior third of eye, the umbilicate punctures mainly distributed at posterior edge in transversal line. Epicranium shiny without microsculpture and with micropunctures between umbilicate punctures, micropunctures denser anteriorly. Gula with two long setae near anterior margin. Labrum with large, apically rounded lobe near middle of anterior margin and with smaller, apically rounded lobe near lateral edge of anterior margin. Antennae with scape gradually thickened, pedicel (0.24) shorter than 2.6 times the length of scape (0.63), pedicel and segment 3 similar in width (0.12), segment 3 longer (0.33) than pedicel (0.24), segment 4 (length 0.22 : width 0.14) longer than wide, segment 5 (0.24 : 0.14) longer than wide, segments 6 to 8 longer than wide and identical measurements (0.20 : 0.14), segment 9 longer than wide (0.16 : 0.13), segment 10 quadrate (0.14 : 0.14), segment 10 longer than wide (0.22 : 0.12); segments 4–11 densely covered with microsetae; scape to segment 3 with black macrosetae lacking a defined pattern, on segment 4 to 10 arranged in one ring in the apical region, on segment 11 in a ring in the middle region and one tuft in the apical region.

Pronotum (Fig. 1) wider than long (PL: 1.53; PW: 1.86), with anterior margin straight, lateral margin slightly concave and hind angles rounded. Disk polished and shiny without microsculpture, with longitudinal row of 7-9 punctures on each side of midline; several punctures on lateral to paramedial row of punctures; rare micropunctures homogeneously distributed. Elytra (Fig. 1) slightly wider than pronotum (EL: 2.08; EW: 1.96) with epipleural ridge; surface polished and shiny, with irregular rows; with black macrosetae.

Legs uniformly covered with glossy black macrosetae; segments 1–4 of protarsus strongly bilobate and with yellowish pale setae ventrally.

Abdomen polished and shiny, uniformly punctate; the first segments more strongly punctate than the last.

Male with broad and moderately deep, median apical emargination on sternum VII (Fig. 4), posterior margin with small carina on lateral edge of emargination. Segment VIII (Figs 6–7) with four internal canals at base of tergum and sternum. Tergum VIII (Fig. 6) with trilobed posterior margin; basal ridge with short median carina. Sternum VIII (Fig. 7) constituted by two lateral plates and one central plate, fused at the base. Central plate with broad, median emargination; the emargination wide apically and strongly narrowed basally, depression margined laterally by longitudinal carinae; each side of depression with additional lateral carinae; basal ridge with longitudinal small grooves and pair of central carinae; between basal and apical carinae is a small carina. Tergum IX (Fig. 5) fused medially and with long black setae. Aedeagus as in Figs. 2–3; parameres symmetric, fused around basal foramen; with broad, deep median apical emargination; ventral side with median cavity between median depression and basal foramen; median depression with small cluster of setae on lateral margin.

Female not known.

#### Habitat.

 From window traps in rainforest.

#### Distribution.

Known from Montagne des Chevaux, French Guiana, 90 m.

#### Etymology.

 This species is named in honor of Dr. Ulrich Irmler of the Institute of Ecosystem Research, Germany.

### Neolindus hermani

Asenjo sp. n.

urn:lsid:zoobank.org:act:2B271F06-AAF9-48EC-B0B7-E904AFA5A527

http://species-id.net/wiki/Neolindus hermani

Figs 8–14 

#### Type material.

 FRENCH GUIANA: Holotype male with labels:"GUYANA FRANCESA: / Montagne des chevaux, / 04°43'N, 52°25'W, 90m[altitude in relation to sea level], / flight intercept trap(glass), / 8.iii.2009, S. Brûlé, / P.H. Dalens, E. Poirier" “Holotype / *Neolindus* / *hermani* Asenjo / Desig. Asenjo, 2011" (DZUP).

#### Paratypes.

5 males with labels: “GUYANA FRANCESA: / Montagne des chevaux, / 04°43'N, 52°25'W, 90m[altitude in relation to sea level], / flight intercept trap(glass), / 2.v.2009, S. Brûlé, / P.H. Dalens, E. Poirier" [without right elytron] (MNHN); “GUYANA FRANCESA: / Montagne des chevaux, / 04°43'N, 52°25'W, 90m[altitude in relation to sea level], / flight intercept trap(glass), / 10.iii.2009, S. Brûlé, / P.H. Dalens, E. Poirier" [without left elytron] (DZUP); “GUYANA FRANCESA: / Montagne des chevaux, / 04°43'N, 52°25'W, 90m[altitude in relation to sea level], / flight intercept trap(glass), / 8.iii.2009, S. Brûlé, / P.H. Dalens, E. Poirier" [without right elytron] (MUSM); “GUYANA FRANCESA: / Réserve Trésor, [around]~225m[altitude in relation to sea level], 04°36'37.6"N, 52°16'44.5"W, / flight intercept trap (glass), / 1.xi.2009, S. Brûlé, P.H. / Dalens, E. Poirier" (MUSM); “GUYANA FRANCESA:The / Nouragues natural reserve, / Saut Pararé, 04°02'17.1"N, 52°40'22.3"W, 80m[altitude in relation to sea level], flight" “intercept trap (glass), / 20.x.2009, S. Brûlé, P.H. / Dalens, E. Poirier" [without elytra] (MUSM). 2 female with labels: “GUYANA FRANCESA: / Montagne des chevaux, / 04°43'N, 52°25'W, 90m[altitude in relation to sea level], / flight intercept trap(glass), / 20.vi.2009, S. Brûlé, / P.H. Dalens, E. Poirier" (MUSM); “GUYANA FRANCESA:The / Nouragues natural reserve, / Saut Pararé, 04°02'17.1"N, 52°40'22.3"W, 80m[altitude in relation to sea level]," “vii.2009, flight intercept trap / (glass), S. Brûlé, P.H. / Dalens, E. Poirier" (MUSM); all paratypes with label “Paratype / *Neolindus* / *hermani* Asenjo / Desig. Asenjo, 2011".

#### Diagnosis.

Among *Neolindus* species, *Neolindus hermani* sp. n. is similar to *Neolindus pastazae*, in having the three triangular lobes on the posterior margin of tergum VIII (Fig. 13) and antennal segment 10 shorter than 9. *Neolindus hermani* sp. n. differs from it by the acute lobe on each side of median apical emargination on sternum VII (Fig. 11) and sternum VIII with a large pair of depressions on each side of the central emargination of the apex (Fig. 14).

#### Description.

Holotype male, BL: 13.75.

**Figures 8–14.**
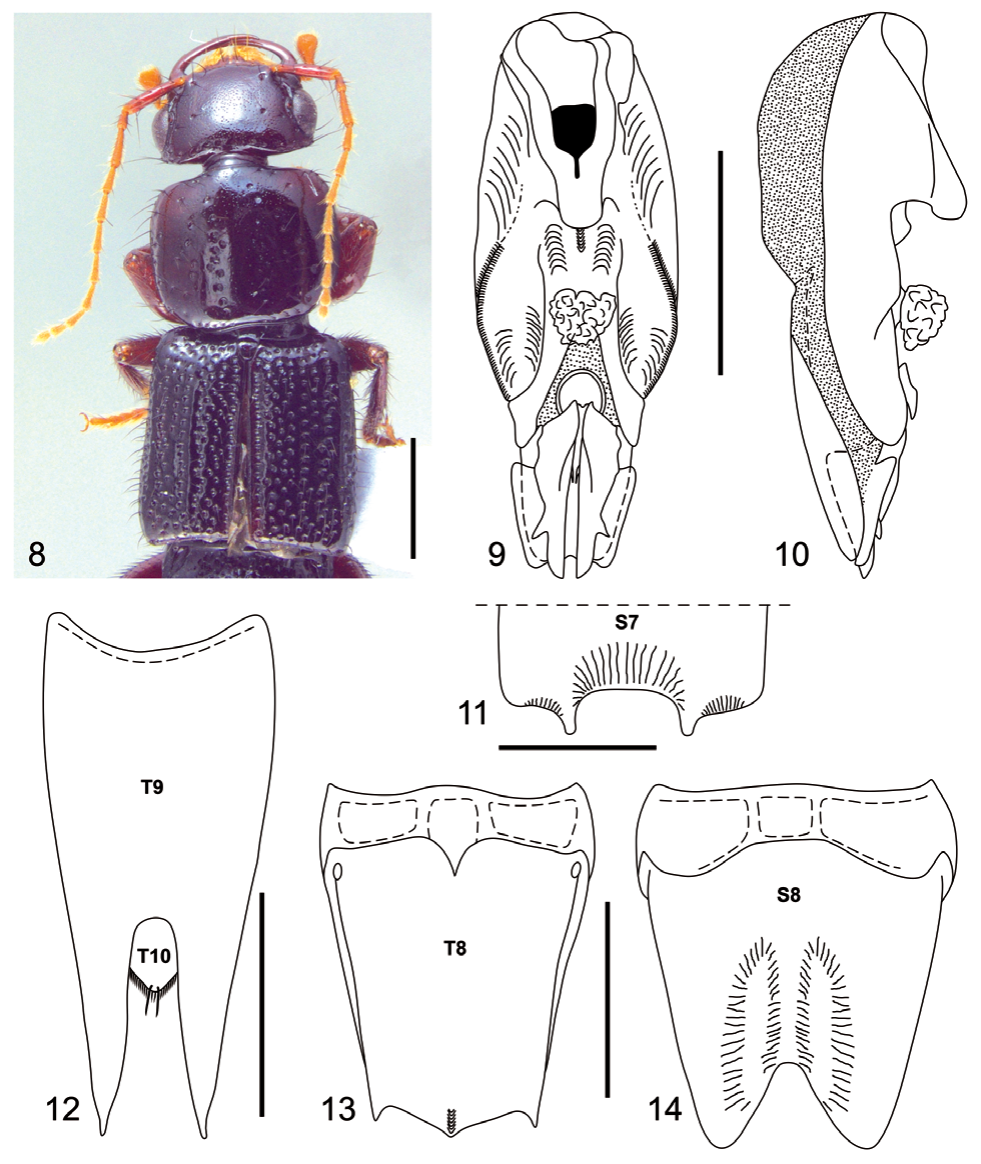
*Neolindus hermani* Asenjo sp. n. holotype male. **8** Adult habitus **9** aedeagus, ventral view **10** aedeagus, lateral view **11** apex of sternum VII (S7), setae omitted **12** tergum IX (T9) and tergum X (T10), setae omitted **13** tergum VIII (T8), setae omitted **14** sternum VIII (S8), setae omitted. Scale bars= 1mm.

Body dark brown (Fig. 8). Mandibles, femora, tibiae and antennal segments 1–2 dark reddish brown; antennal segments 3–11 reddish brown to yellow; all tarsi paler.

Head and pronotum moderately flattened dorsoventrally. Head (Fig. 8) wider (HW: 1.90) than long (HL: 1.22), with acute hind angles. Head disk with umbilicate punctures each carrying a black macroseta and one trichobothrium on lateral side of vertex near anterior third of eye. The umbilicate punctures mainly distributed at posterior edge in transversal line. Epicranium shiny without microsculpture and with micropunctures between umbilicate punctures, micropunctures homogeneously distributed. Gula with transverse cluster of numerous setae near anterior margin. Labrum with large, apically rounded lobe near middle of anterior margin and with smaller, apically rounded lobe near lateral edge of anterior margin. Antennae with scape gradually thickened, pedicel (0.20) shorter than 3.8 times the length of scape (0.76), scape (0.14) wider than pedicel (0.11), segments 3–11 longer than wide and with identical width (0.10), segment 3 (0.47) longer than pedicel (0.20), length segments 4 and 8 (0.31), length segments 5 and 6 (0.35), length segment 7 (0.33), length segment 9 (0.24), length segments 10 and 11 (0.20); segments 3–11 densely covered with microsetae; scape and pedicel with black macrosetae lacking a defined pattern, on segment 3 to 10 arranged in one ring in the apical region, on segment 11 in a ring in the middle region and one tuft in the apical region.

Pronotum (Fig. 8) wider than long (PL: 1.82; PW: 2.12), with anterior margin straight, lateral margin slightly concave and hind angles rounded. Disk polished and shiny without microsculpture; with longitudinal row of 8–11 punctures on each side of midline; several punctures on lateral to paramedial row of punctures; micropunctures homogeneously distributed. Elytra (Fig. 8) slightly wider than pronotum (EL: 2.45; EW: 2.33) with epipleural ridge; surface polished and shiny, with irregular rows; with black macrosetae.

Legs uniformly covered with glossy black macrosetae; segments 1–4 of protarsus strongly bilobate and with yellowish pale setae ventrally.

Abdomen polished and shiny, uniformly punctate; the first segments more strongly punctate than the last. Segments VII and VIII with microsculpture between punctures.

Male with broad and deep median apical emargination on sternum VII (Fig. 11), posterior margin with lobe on lateral edge of emargination; surface adjacent to emargination with shallow median depression. Segment VIII (Figs 13–14) with four internal canals at base of tergum and sternum. Tergum VIII (Fig. 13) with three triangular, apically acute lobes on posterior margin, apex of central lobe longer than lateral lobes; basal ridge with short median carina; surface with slightly midlongitudinal carina on apical portion of median lobe; Sternum VIII (Fig. 14) with large pair of depressions on each side of central emargination of apex. Tergum IX (Fig. 12) fused medially and with long black setae. Aedeagus as in Figs 9–10; parameres symmetric and fused to median lobe; with broad, deep median apical emargination; ventral side with median carina in front of basal foramen; apex of median lobe with many sclerites exposed.

Female with characters of head, pronotum, and elytra as described for male. Abdominal sterna VII and VIII with posterior margin emarginated.

#### Habitat.

 From window traps in rainforest.

#### Distribution.

Known from Montagne des Chevaux (90m), Nouragues natural reserve-Saut Pararé (80m) and Réserve Trésor (225m) from French Guiana.

#### Etymology.

 This species is named in honor of Dr. Lee Herman of the American Museum of Natural History, USA.

##### Key to the males of Neolindus species

The Peruvian species *Neolindus amazonicus*, *Neolindus hanagarthi* and *Neolindus peruvianus* described for Irmler 1981 are excluded from the key because they are known only from females.

**Table:** 

1	Head with one pair of trichobothria (Fig. 64 in Herman 1991)	2
–	Head with two pairs of trichobothria (Fig. 78 in Herman 1991)	26
2(1)	Pronotum longer than wide	3
–	Pronotum wider than long (Fig. 64 in Herman 1991)	10
3(2)	Tergum VIII with posterior margin rounded or truncate (Fig. 122 & 148 in Herman 1991)	4
–	Tergum VIII with posterior margin emarginate (Fig. 136 in Herman 1991)	7
4(3)	Antennal segments 3 to 11 with dense pubescence. Peru (Huánuco)	*Neolindus verhaaghi*
–	Antennal segments 4 to 11 with dense pubescence	5
–	Antennal segments 5 to 11 with dense pubescence	6
5(4)	Abdominal tergum VIII (Fig. 122 in Herman 1991) with posterior margin rounded and without internal canals at base, segment III without paratergites; aedeagus with apex of median lobe pointed (Fig. 119 in Herman 1991). Costa Rica (Puntarenas)	*Neolindus incanalis*
–	Abdominal tergum VIII (Herman 1991; Fig. 140) with posterior margin truncate and with internal canals at base, segment III with one pair of paratergites; aedeagus with apex of median lobe emarginate (Fig. 141 in Herman 1991). Peru (Cuzco)	*Neolindus cephalochymus*
6(4)	Tergum VIII with posterior margin rounded (Fig. 127 in Herman 1991). Bolivia (Beni), Brazil (Pará)	*Neolindus brewsterae*
–	Tergum VIII with posterior margin sinuotruncate (Fig. 144 in Herman 1991). Brazil (Distrito Federal)	*Neolindus agilis*
7(3)	Head without midlongitudinal carina at anterior margin	8
–	Head with midlongitudinal carina at anterior margin	9
8(7)	Antennal segment 2 longer than 3, segments 5–11 with dense pubescence. Colombia (Amazonas), Brazil (Pará)	*Neolindus densus*
–	Antennal segment 2 shorter than 3, segments 4–11 with dense pubescence. Peru (Ucayali, Madre de Dios)	*Neolindus punctiventris*
9(7)	Body length about 9.00 mm; tergum VIII with moderately deeply to shallowly emarginate posterior margin (Fig. 159 in Herman 1991). Ecuador (Napo, Pichincha)	*Neolindus retusus*
–	Body length about 6.00 mm; tergum VIII with feebly emarginate posterior margin (Fig. 165 in Herman 1991). Ecuador (Pichincha)	*Neolindus procarinatus*
10(2)	Tergum VIII with posterior margin rounded or truncate (Fig. 178 & 215 in Herman 1991)	11
–	Tergum VIII with posterior margin emarginate, lobed or trilobed (Figs. 203, 157 & 206 in Herman 1991)	18
11(10)	Tergum IX with base fused medially (Fig. 180 in Herman 1991)	12
–	Tergum IX with base divided medially (Fig. 171 in Herman 1991)	13
12(11)	Antennal segment 2 longer than 3. Costa Rica (Puntarenas)	*Neolindus cuneatus*
–	Antennal segment 2 shorter than 3. Ecuador (Napo)	*Neolindus milleri*
13(11)	Antennal segments 3 to 11 with dense pubescence; median orifice of median lobe of aedeagus with the sclerites hidden (Fig. 170 in Herman 1991)	14
–	Antennal segments 4 to 11 with dense pubescence; median orifice of median lobe of aedeagus with the sclerites exposed (Fig. 214 in Herman 1991)	15
14(13)	Aedeagus on ventral surface with median apical carina on median lobe (Fig. 170 in Herman 1991). Panama (Panama)	*Neolindus campbelli*
–	Aedeagus on ventral surface without median apical carina on median lobe (Fig. 174 in Herman 1991). Panama (Panama)	*Neolindus apiculus*
15(13)	Tergum VIII with posterior margin rounded (Fig. 183 in Herman 1991); aedeagus without median hole on ventral surface (Fig. 186 in Herman 1991); antennal segment 2 shorter than 3	16
–	Tergum VIII with posterior margin truncate (Fig. 215 in Herman 1991); aedeagus with median hole on ventral surface (Fig. 214 in Herman 1991); antennal segment 2 and 3 subequal in length. Ecuador (Napo)	*Neolindus dichymus*
16(15)	Aedeagus without setae on ventral surface (Fig. 186 in Herman 1991). Brazil (Pará)	*Neolindus lodhii*
–	Aedeagus with setae on ventral surface (Fig. 198 in Herman 1991)	17
17(16)	Sternum VIII with shallow apical emargination; emargination about one-fifteenth of length of sternum (Fig. 199 in Herman 1991). Brazil (Pará)	*Neolindus sinuatus*
–	Sternum VIII with deep apical emargination; emargination about one-fifth of length of sternum (Fig. 195 in Herman 1991). Costa Rica (Heredia), Panama (Panama, Canal Zone)	*Neolindus basisinuatus*
18(10)	Aedeagus without median hole on ventral surface (Fig. 156 in Herman 1991)	19
–	Aedeagus with median hole on ventral surface (Fig. 210 in Herman 1991)	22
19(18)	Antennal segment 2 longer than 3; gula with two setae	20
–	Antennal segment 2 shorter than 3; gula with transverse cluster of setae	21
20(19)	Tergum VIII with posterior margin lobed and middle of basal ridge carinated (Fig. 203 in Herman 1991); tergum IX with the base divided medially (Fig. 201 in Herman 1991). Brazil (São Paulo)	*Neolindus unilobus*
–	Tergum VIII with posterior margin emarginated and middle of basal ridge not carinated (Fig. 157 in Herman 1991); tergum IX with the base fused medially (Fig. 155 in Herman 1991). Ecuador (Pichincha)	*Neolindus bullus*
21(19)	Tergum VIII without carina in middle of basal ridge (Fig. 2F in Irmler 2011); antennal segment 10 shorter than 9, segments 3–11 with dense pubescence (Fig. 2A inIrmler 2011).Ecuador (Tungurahua)	*Neolindus pastazae*
–	Tergum VIII with middle of basal ridge pointed (Fig. 206 in Herman 1991); antennal segments 9 and 10 subequal in length, segments 4–11 with dense pubescence. Panama (Chiriqui)	*Neolindus punctogularis*
22(18)	Tergum VIII with middle of basal ridge carinate (Fig. 221 in Herman 1991)	23
–	Tergum VIII with middle of basal ridge pointed	24
23(22)	Gula with two setae; antennal segments 2 and 3 subequal in length. Ecuador (Pichincha)	*Neolindus bidens*
–	Gula with four setae; antennal segment 2 shorter than 3. Brazil (São Paulo, Rio de Janeiro)	*Neolindus schubarti*
24(22)	Sternum VIII not divided (Fig. 14)	25
–	Sternum VIII divided into three plates, one central and two lateral (Fig. 7). French Guiana	*Neolindus irmleri* sp. n.
25(24)	Gula with two setae; antennal segments 9 and 10 subequal in length and 4 to 11 with dense pubescence; aedeagus with setae on ventral surface (Fig. 224 in Herman 1991). Brazil (Pará)	*Neolindus religans*
–	Gula with transverse cluster of setae; antennal segment 10 shorter than 9 and 3 to 11 with dense pubescence; aedeagus without setae on ventral surface (Fig. 9). French Guiana	*Neolindus hermani* sp. n.
26(1)	Tergum VIII with posterior margin rounded (Fig. 82 in Herman 1991)	27
–	Tergum VIII with posterior margin emarginate, trilobed (Fig. 109 & 117 in Herman 1991)	31
27(26)	Aedeagus with apex of median lobe pointed (Fig. 81 in Herman 1991)	28
–	Aedeagus with apex of median lobe emarginate (Fig. 85 in Herman 1991)	30
28(27)	Sternum VIII with surface of apex of internal canals unmodified. Venezuela (Trujillo)	*Neolindus plectrus*
–	Sternum VIII with depressions in the surface of apex of internal canals (Fig. 92 in Herman 1991)	29
29(28)	Aedeagus distinctive and without transversal carina or process on ventral surface (Fig. 105 in Herman 1991). Venezuela (Aragua)	*Neolindus rudiculus*
–	Aedeagus distinctive and with transversal carina or process on ventral surface (Fig. 93 in Herman 1991). Colombia (Valle del Cauca)	*Neolindus pumicosus*
30(27)	Antennal segment 2 longer than 3. Ecuador (Napo)	*Neolindus parallelus*
–	Antennal segments 2 and 3 subequal in length. Venezuela (Aragua)	*Neolindus brachiatus*
31(26)	Pronotum longer than wide; aedeagus with apex of median lobe pointed (Fig. 113 in Herman 1991)	2
–	Pronotum wider than long; aedeagus with apex of median lobe emarginate (Fig. 152 in Herman 1991). Brazil (Pará)	*Neolindus hamatus*
32(31)	Elytra shorter than pronotum; tergum VIII with posterior margin emarginate (Fig. 109 in Herman 1991). Ecuador (Aragua)	*Neolindus lirellus*
–	Elytra longer than or as long as pronotum; tergum VIII with posterior margin trilobed (Fig. 117 in Herman 1991). Ecuador (Napo)	*Neolindus prolatus*

## Supplementary Material

XML Treatment for Neolindus irmleri

XML Treatment for Neolindus hermani
